# Bayesian spatio-temporal distributed lag modeling for delayed climatic effects on sparse malaria incidence data

**DOI:** 10.1186/s12874-021-01480-x

**Published:** 2021-12-20

**Authors:** Chawarat Rotejanaprasert, Nattwut Ekapirat, Prayuth Sudathip, Richard J. Maude

**Affiliations:** 1 Department of Tropical Hygiene, Faculty of Tropical Medicine, Mahidol University, Ratchathewi, Bangkok, 10400 Thailand; 2 Mahidol-Oxford Tropical Medicine Research Unit, Faculty of Tropical Medicine, Mahidol University, Bangkok, Thailand; 3 Division of Vector Borne Diseases, Department of Disease Control, Ministry of Public Health, Nonthaburi, Thailand; 4 Harvard T.H. Chan School of Public Health, Harvard University, Cambridge, MA USA; 5 Centre for Tropical Medicine and Global Health, Nuffield Department of Medicine, University of Oxford, Oxford, UK; 6 The Open University, Milton Keynes, UK

**Keywords:** Spatiotemporal, Malaria, Bayesian, Lag effect, Weather

## Abstract

**Background:**

In many areas of the Greater Mekong Subregion (GMS), malaria endemic regions have shrunk to patches of predominantly low-transmission. With a regional goal of elimination by 2030, it is important to use appropriate methods to analyze and predict trends in incidence in these remaining transmission foci to inform planning efforts. Climatic variables have been associated with malaria incidence to varying degrees across the globe but the relationship is less clear in the GMS and standard methodologies may not be appropriate to account for the lag between climate and incidence and for locations with low numbers of cases.

**Methods:**

In this study, a methodology was developed to estimate the spatio-temporal lag effect of climatic factors on malaria incidence in Thailand within a Bayesian framework. A simulation was conducted based on ground truth of lagged effect curves representing the delayed relation with sparse malaria cases as seen in our study population. A case study to estimate the delayed effect of environmental variables was used with malaria incidence at a fine geographic scale of sub-districts in a western province of Thailand.

**Results:**

From the simulation study, the model assumptions which accommodated both delayed effects and excessive zeros appeared to have the best overall performance across evaluation metrics and scenarios. The case study demonstrated lagged climatic effect estimation of the proposed modeling with real data. The models appeared to be useful to estimate the shape of association with malaria incidence.

**Conclusions:**

A new method to estimate the spatiotemporal effect of climate on malaria trends in low transmission settings is presented. The developed methodology has potential to improve understanding and estimation of past and future trends in malaria incidence. With further development, this could assist policy makers with decisions on how to more effectively distribute resources and plan strategies for malaria elimination.

## Background

Among the parasitic diseases worldwide, malaria is one of the most prevalent [1]. It is caused by protozoan parasites of the genus *Plasmodium*. In the Greater Mekong Subregion (GMS), the predominant species are *P. falciparum* and *P. vivax.* As the species most likely to cause severe complications and death, and lacking a dormant liver stage, *P. falciparum* has been the main focus of elimination plans among countries in the region. Migration and agricultural activities along the country’s borders have been studied and found to be associated with malaria risk. Either side of the Thailand-Myanmar border has consistently been an area with relatively high transmission for many years [2]. With intense efforts to eliminate malaria by 2030, the number of *P. falciparum* incidence has markedly decreased in recent years across the GMS [3]. This includes on the Thai side [4] and in Kayin State in Myanmar [5]. In many areas of the Greater Mekong Sub-region, malaria is now narrowed down to small foci of transmission and there has been an intensification of efforts by National Malaria Control Programs and partners to better understand and stop residual transmission in order to achieve elimination.

The malaria parasite is transmitted from human to human via the bite of infected female mosquitoes of the genus *Anopheles.* The malaria case distribution and dynamics have been found to be closely related to environmental factors, particularly in high transmission areas in Subsaharan Africa [6]. Studies have shown that both mosquito species and malaria parasites are very sensitive to weather conditions [6–11]. For example, moderate rainfall can create mosquito breeding sites while temperature affects the rate of development of the mosquito larvae and influences mosquito survival rates [12, 13]. In addition, higher temperatures also accelerate multiplication of the *Plasmodium* parasites inside the vectors [14]. Suitable climatic conditions can create circumstances appropriate for malaria transmission in endemic areas. Therefore, how malaria incidences change as a result of climatic variability is an important understanding for effective malaria control planning and activities [15]. The relationship between climate and malaria incidence is complex with inconsistent findings perhaps due to regional variations and limited availability of suitable methods [16].

To investigate the relationship between meteorological variables and malaria incidence, it is sensible to account for biology of the transmission process. It has been shown that the relationship between climate and malaria is stronger for *P. falciparum* than for *P. vivax* [17]. This is likely to be due to a proportion of *P. vivax* cases being recurrences of dormant liver stage parasites (not found in falciparum infections) rather than being transmitted by mosquitoes. Epidemiologically there is a lag between changes in environmental factors and malaria transmission due to both the life cycle of mosquitoes (only mature adult females transmit malaria) and incubation of parasites in the mosquitoes (which are transmitted once they develop into sporozoites). This forms a lagged time-varying distribution of the association. Therefore, the lag effect should then be addressed in analyses. To account for the lagged correlation, distributed lag nonlinear modeling (DLNM) has been shown to be a valuable and effective tool [18–20].

Distributed lag models (DLMs) have been widely applied in epidemiological studies to estimate associations between environmental variables and health outcome of interest at different lagged periods (for example please see [20–23]). Although the use of DLMs to quantify the lag effect of climatic factors and malaria incidences has been used (e.g., [10, 11, 16, 24]), currently few studies have investigated the possibility of spatio-temporal variation in health outcomes within the lagged regression modeling. Contributors to such spatial variation can include exposure characteristics which could be spatially varying environmental composition and spatial differences in exposure measurements [25]. To account for spatial variation, geostatistical point process methods have been used to introduce spatial correlation between lagged regression parameters corresponding to different discrete spatial regions while defining correlation as a function of distance between region centroids [26]. However, this distance may be inappropriate when the spatial regions are oddly shaped and/or their sizes vary greatly [27]. Recently a spatially varying distributed lag model at individual level has been developed with application to an air pollution and term low birth weight study [25]. However, since the number of *P. falciparum* malaria cases of countries in GMS has decreased and distribution of cases is increasingly sparse as areas progress towards elimination, the excess of zero cases is another analytical challenge that has not been considered in previous modeling.

Excessive zeros commonly occur and are often encountered in biostatistical and epidemiological research especially for nearly eliminated and neglected tropical diseases. Based on different data generation mechanisms, the large number of zeros can lead to overdispersion caused by a disagreement between the data and the assumed distribution. This can result in having more zeroes in our data than the proposed distribution could reasonably explain. The zero valued data should not be ignored and dropped from the analysis as they often provide important information regarding the process. In addition, having a large proportion of zeros could indicate important characteristics of the data or condition under study. Thus, probabilistic models that are capable of handling excessive zeros should be considered. Excessive-zero modeling has been frequently adopted in the disease mapping literature (see for example [28–31]). To account for the temporal lagged effect, an autoregressive integrated moving average (ARIMA) model has been used to model correlated residual in relative malaria risk [32]. However, that method does not directly address the collinearity in climate covariates which is a known issue [33, 34] and could be accounted for using DLMs.

Therefore, in this study we aimed to develop a framework to estimate the temporal lag effect of climatic factors on *P. falciparum* malaria incidence in Thailand. Whereas the relationship of multiple malaria species has been investigated [35–37], in this work we focused on the association of the single species with climatic variables which is a current concern of disease control and elimination in the area. The sub-district data were from passive surveillance at level which there have not been analyses well performed at this fine spatial scale. We described research motivation in the context of malaria elimination and provided the basic model used in spatio-temporal analysis. The concept of DLMs as a foundation of our methodology to describe a lagged effect of climate on malaria incidence was introduced. Then the additional complexity of the zero-inflation issue in our data set and spatio-temporal distributed lagged modeling for zero inflated data was described. The robustness of the proposed framework was examined through a simulation study and a case study to estimate the delayed effect of environmental variables on malaria incidence in Tak Thailand along the Thailand-Myanmar border, one of the areas with the highest malaria burden in Thailand. Finally, analytic challenges and developments are further discussed.

## Methods

### Study design

In this study we first developed a methodology to investigate the lagged distribution of climatic effects on *P. falciparum* malaria cases in western Thailand. Then a simulation study was conducted to examine the performance of the models under various parameter conditions and assessed using a number of evaluation metrics. A case study with retrospective analysis design was also provided as a real data example of lagged effects on malaria cases in an elimination setting along the Thailand-Myanmar border. Weekly malaria incidence data of Tak in 2016 obtained from the Bureau of Vector Borne Disease at the Ministry of Public Health were used to demonstrate the application of the proposed model. To quantify the association with climatic factors, data on average temperature, relative humidity and total rainfall were considered in this study and collected from the Meteorological Department from five weather stations across the region during the study period.

### Modeling for spatially sparse health data

**Fig. 1:**
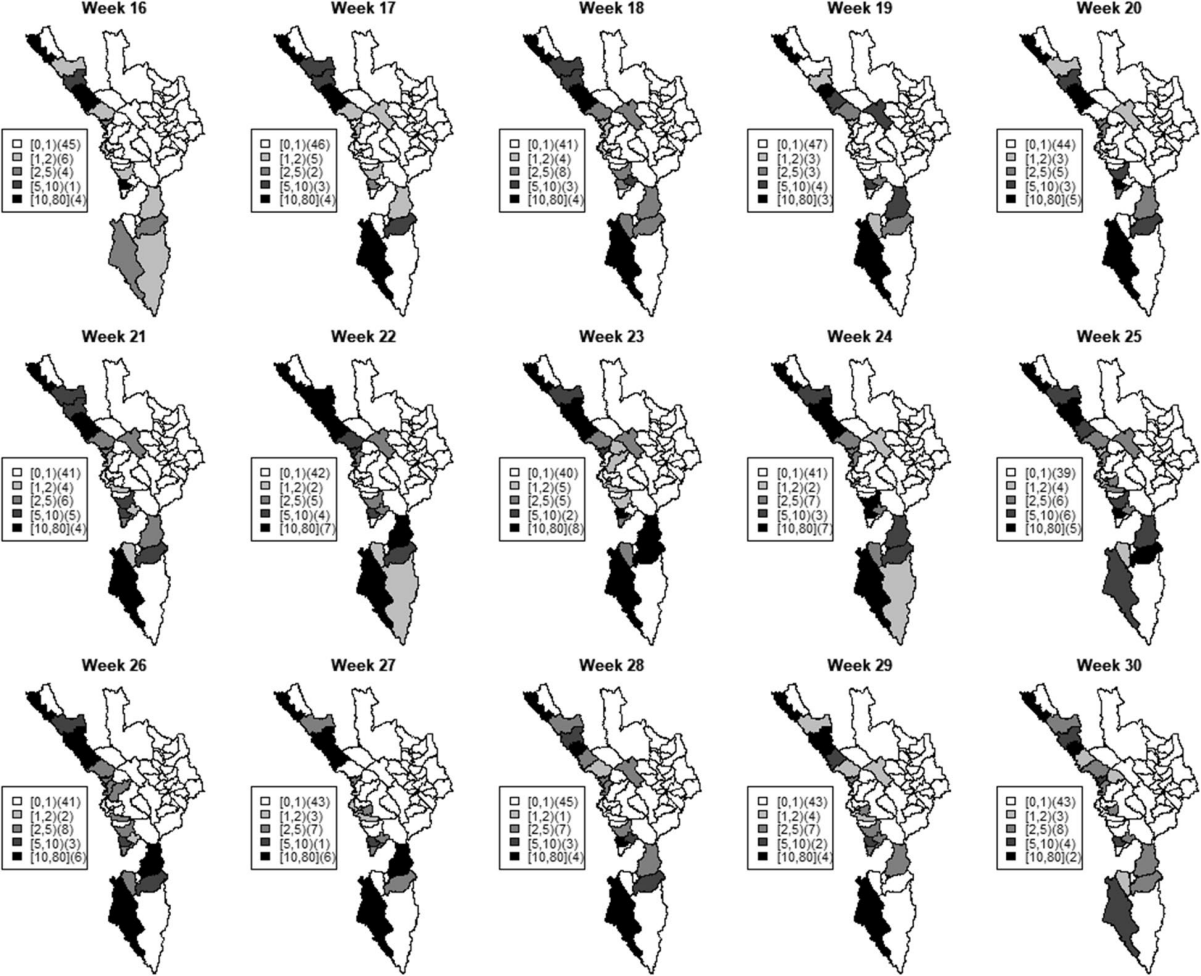
Maps of sub-district level *P. falciparum* malaria incidence (as indicated in each interval under different colors) in Tak province during the rainy season (weeks 16–30) in 2016

**Fig. 2:**
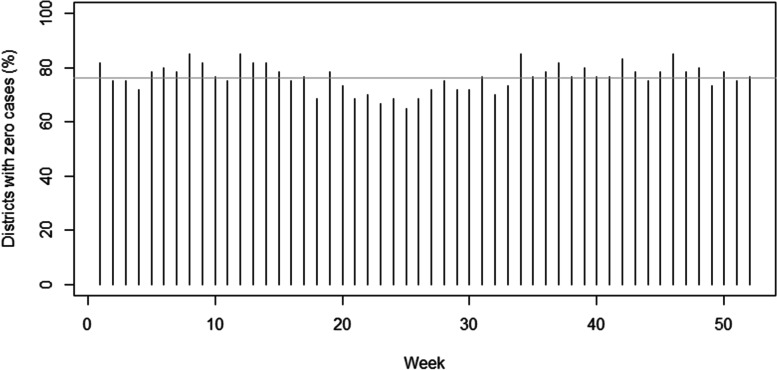
Plot of weekly percentages of districts without *P. falciparum* malaria cases in Tak province in 2016. The grey line represents the overall mean of 76.28%

The analysis of malaria incidence over space and time has received considerable attention due to growing demand for reliable estimation in order to plan effective control activities. Modeling incidence from surveillance health data indexed at a fine spatial resolution poses specific statistical problems due to the sparse nature of the data, especially in elimination settings. Figure 1 presents the number of *P. falciparum* malaria cases at sub-district level (as indicated in each interval under different colors) in Tak province in 2016 during weeks 16–30 which was the rainy season, the period of high malaria transmission in the region due to weather suitability. Figure 2 shows histograms of malaria incidence in the same study population. Both figures present the sparseness in our data set. In general, estimates of small areas can be sensitive to sampling variation due to relative population size to rare disease incidence among areas [38, 39]. Model-based methods have been developed to address the variability issue when mapping health outcomes at small geographical resolutions. These methods help to decrease the variability in small area estimates in sparse health data using spatial smoothing, which allows study units to integrate strength from surrounding regions to yield a more stable estimate in each area (see examples [39–41]).

The small area modeling has been widely applied on disease mapping with an assumption of Poisson likelihood [39, 42]. However, ordinary count distributions, such as regular Poisson distribution cannot adequately capture the variation due to the sparse nature of the malaria cases in our study population which also produce excessive zero cases in the data set as depicted in Figs. 1 and 2. This situation occurs primarily in disease elimination areas in which there is an abundance of zeros coupled with a heterogeneous distribution of positive counts, as these features impose competing influences on the model and potentially become problematic. In the Poisson case, for example, the high proportion of zeros tends to lower the mean parameter. While alternative count distributions, such as the negative binomial, can add flexibility by incorporating a separate heterogeneity parameter, these distributions do not always ensure adequate fit [43] and neglect of excess zeroes will bias the estimation of parameters [44].

### Spatio-temporal modeling for excessive zero malaria cases

The excessive amount of zero incidence is an analytical challenge of eliminating disease modeling. One possible explanation for those zero cases is that they occur in spatial unit where malaria transmission is not suitable to occur during the study period. This can happen for various epidemiological reasons such as vector habitat or environmental unsuitability or effective interventions. To address excessive zeros in our data, there is a broad class of spatio-temporal models to accommodate both zero inflation and nonzero counts. The two widely used approaches are hurdle and zero-inflation models. One limitation of standard models for count data is that the two types of data, zeros and the positive integers are considered to be from the same data-generating process. However, f*or hurdle models*, these two mechanisms are not assumed to be the same. The basic idea is that a probability distribution governs the binary index of whether a count outcome has a zero or positive integer. If the realization is positive, the hurdle model is crossed, and the conditional distribution of the positive counts is formed by a truncated-at-zero count data model. On the other hand, *with zero-inflated models,* the response variable is modelled as a mixture of a Bernoulli distribution (otherwise called a point mass at zero) and a base count distribution. However, for our malaria incidence data, it is perhaps more sensible to assume that our observed zero counts were from both mechanisms which could also account for asymptomatic cases. Hence, we would consider zero-inflation rather than hurdle modeling in this study.

Zero-inflation models can be seen as mixture modeling with a point mass at zero with a base distribution for the positive outcomes [30, 45]. Letting *Y*_*it*_ denote a random variable representing the malaria case count in sub-district *i* in week *t*, the generic structure of the zero-inflation model is given by

$${Y}_{it}={y}_{it}\sim \left(1-\pi \right){I}_{\left({y}_{it}=0\right)}+\pi f\left({y}_{it}|{\mu}_{it},\boldsymbol{\theta} \right){I}_{\left({y}_{it}>0\right)}$$

where *I*_(.)_ denotes as the indicator function; *π* = *p*(*Y*_*it*_ > 0) is the probability of a non-zero malaria case; *f*(*y*_*it*_| *μ*_*it*_, ***θ***) is the base probability distribution with mean *μ*_*it*_ conditioned on all parameters ***θ*** in the model. The spatio-temporal zero-inflation model specified above partitions the zeros into two types: structural or inaccessibility zeros. Hence, the true (inaccessibility) zeros part can, for instance, be represented as those that occur because they were not susceptible to malaria infection (e.g. residence in areas unsuitable for transmission). The structural part is to generate chance or sampling zeros which form those that occur by chance among those with susceptibility. So *π* can be expressed as the “at-risk” probability which can also be interpreted as the probability of belonging to an at-risk sub-population [46, 47].

### Lagged effects of potential climate factors on malaria incidence

The association between malaria incidence and different environmental factors is complex with inconsistent findings between studies. Besides the variations due to excessive zeros which can pose an analytical challenge which was addressed in the previous section, the inconsistent evidence of the association may also result from invalid statistical assumptions including from the misspecification of the single fixed lag. The comprehensive lag pattern for climatic factors which has been thoroughly examined previously should be investigated. Thus, lagged modeling may have the potential to better understand the relationship and improve forecasting of changes in malaria incidence which would help public health authorities on how to more effectively plan and distribute resources for malaria elimination.

Modeling the relationship with environmental exposures then requires the additional lag dimension of an exposure–incidence relationship, describing the time series of the effect. Thus, we further extended the spatiotemporal zero-inflated modeling in the previous section to account for the delayed effects. Distributed lag modeling has been developed and applied to many areas (please see examples in [18, 19, 21, 22, 48]). A general specification to describe the lagged structure can be expressed as

$$h\left({\mu}_{it}\right)={\beta}_0+{\sum}_{l=1}^Lg\left({x}_{it-l};{\boldsymbol{\beta}}_l\right)+{\boldsymbol{\delta}}_{it}$$

where *L* is the maximum lag. *μ*_*it*_ is the mean malaria incidence of each sub-district *i* and week *t*, from an assumed base distribution, i.e. *E*(*Y*_*it*_) = *μ*_*it*_. *h*(.) is the link function of mean incidence and *g*(.) represents a function of the association between malaria incidence and exposures, ***x***_*it*_, with coefficient parameter vector ***β***_*l*_. The variable ***δ***_*it*_ could include random effect terms and other risk factors with linear effects specified by the related coefficients. This specification allows the effect of a single risk factor to be modeled over a period of time, using various parametric forms to describe the distributed contributions at different time lags.

This class of models has been extensively used to assess the lagged effects of environmental factors which can be done through the choice of a basis function [12]. The related basis functions comprise a set of completely known transformations of the original exposure variable that generate a new set of variables, termed basis variables though specification of *g*(.). The simplest formulation is an unconstrained structure, specified in a form of the inclusion a parameter for each lag using direct linear summation [21, 49]. However, this estimation of effects at each time lag can be very unstable and in turn inflate variance due to the collinearity between exposures at lag times [50]. Then some conditions can be imposed to improve the estimation precision of the distributed lag association. For instance, a constant association within lagged time intervals [23], or a smooth temporal relation using continuous mean functions, e.g., polynomial transformations [21, 51] or splines [22] can be assumed. This family of model specifications can be applied in which the formulation of the lags along time series is modeled with an appropriate basis function. Thus, an expression of the constrained model can be generally defined as *g*(***x***_*it*_; ***β***_*l*_) = ∑_*l*_*β*_*l*_*x*_*it* − *l*_, where the coefficient vector is also a linear combination of basis variables. Hence, ${\beta}_l={\sum}_m^M{B}_{lm}{a}_m$ where *B*_*lj*_ is a member in the (*L* + 1) × *M* matrix of basis variables with the particular functional bases with degree of freedom *M* to the time lag *l* vector and ***a***_*m*_, corresponding coefficient vector for the basis function. There are variants of the basis function as mentioned. For instance, the matrix can be defined as a diagonal matrix for the unconstrained model, or a series of polynomials or spline mean functions of period *l* for distributed modeling to explain the effect as a smoothed association along lagged time periods.

### Spatio-temporal distributed lag modeling for sparse areal malaria incidence data

To investigate the association with climatic factors, we propose a spatio-temporal distributed lag space-time model for excessive zeros in our study population. To capture the variability in the positive count data, we consider a Poisson distribution as a standard assumption for areal disease mapping [39, 42]. Although alternatives such as Negative Binomial distribution have also been used to manage dispersion problems, the Poisson likelihood with random effect terms can account for the extra variation in space-time disease mapping [52]. Therefore, the proposed model can be expressed as

$${\displaystyle \begin{array}{l}p\left({Y}_{it}=0\right)=\left(1-{\pi}_i\right)+{\pi}_i{e}^{-{\mu}_{it}}\\ {}p\left({Y}_{it}>0\right)={\pi}_i\frac{{\mu_{it}}^{y_{it}}}{y_{it}!},{\mu}_{it}>0\\ {}i=1,\dots, I;t=1,\dots, T\\ {}\log \left({\mu}_{it}\right)=\log \left(\mathrm{po}{\mathrm{p}}_i\right)+{\delta}_{it}+{\sum}_k^K{\sum}_l^L{\beta}_l{x}_{kit-l}\\ {}{\beta}_l={\sum}_m^M{B}_{lm}{a}_m;{\delta}_{it}={\beta}_0+{u}_i+{\lambda}_t\end{array}}$$

The conditional mean malaria incidence *μ*_*it*_ is linked to the linear predictor through a logarithm function as the conical link function for the exponential family with the offset set as the population on the log scale in each spatial unit. Then the conditional mean and variance of the zero-inflation Poisson (ZIP) model are *E*(*Y*_*it*_) = *π*_*i*_*μ*_*it*_ and *Var*(*Y*_*it*_) = *π*_*i*_*μ*_*it*_[1 + (1 − *π*_*i*_)*μ*_*it*_]. *β*_0_ is the common intercept *B*_*lm*_ is generated from a natural spline basis matrix with degree of freedom *J* to the delay *l* vector and *a*_*m*_ a vector of corresponding parameters specific for distributed lag coefficients.

For parameter estimation, we applied a fully Bayesian approach in which prior distributions for all parameters in the model need to be assumed. In general, it is important to include full random effects in space and time dimensions with interactions. However, in this case we aim to reduce the variation in the estimation and overly saturated specification could lead to severe identifiable estimates which might result in an increase in variance instead. Therefore, we rely in our analysis on a parsimonious specification using separate spatial and temporal random terms. To incorporate the spatial correlation structure and borrowing of information across neighboring sub-districts, the spatially structured effect, *u*_*i*_, is assumed to follow the intrinsic conditional autoregressive (ICAR) model proposed by Besag et al. [53]. The spatial correlation is introduced by defining $\left[{u}_i|{u}_j,i\ne j,{\tau}_i^{-1}\right]\sim N\left(\overline{u_i},{\tau}_i^{-1}\right),$ where $\overline{u_i}$ is the weighted average and *τ*_*i*_ is its precision parameter. They are defined as $\overline{u_i}=\frac{\sum_j{u}_j{w}_{ij}}{\sum_j{w}_{ij}},$ and ${\tau}_i^{-1}=\frac{\tau_u^{-1}}{\sum_j{w}_{ij}}$. The ICAR model assumes maximum spatial correlation with a binary weighted matrix *w*_ij_ = 1 for neighboring districts and *w*_ij_ = 0 otherwise, is taken. The temporal effect *λ*_*t*_ is modelled by a first order random walk process, where each week is influenced by variability of the previous except the first. In general, a random walk is assumed to have a prior Gaussian distribution with mean as the previous time point which can be either positive or negative [39, 54, 55]. Then the temporal trend can be expressed as ${\lambda}_t\sim Normal\left({\lambda}_{t-1},{\tau}_{\lambda}^{-1}\right),t>0;{\lambda}_t\sim Normal\left(0,{\tau}_{\lambda}^{-1}\right),t=0$. The prior distribution of all precision parameter was assumed as a non-informative distribution [39, 42, 56, 57].

### Simulation study

**Fig. 3:**
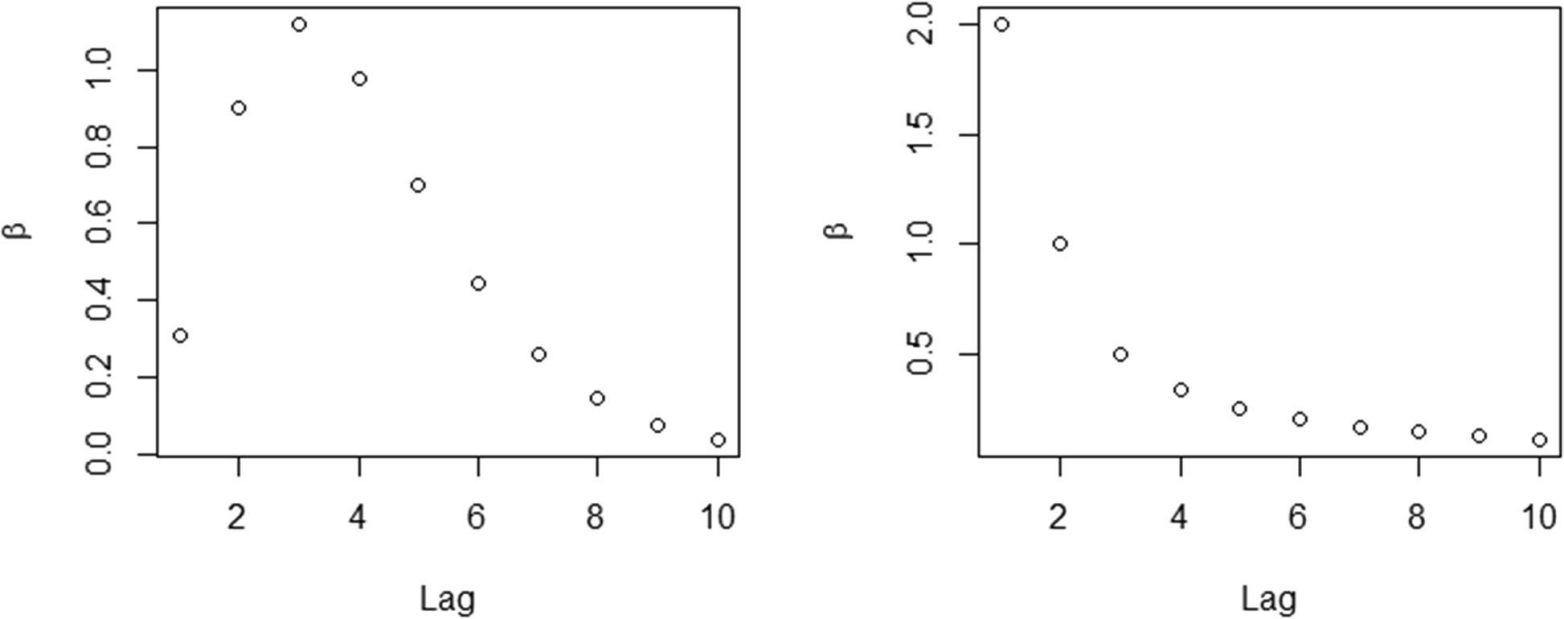
Plots of coefficients with lagged effect (*β*_*l*_) of two assumed situations used in the simulation study. Lag shown in weeks

To evaluate the performance of the proposed spatio-temporal models, we conducted a study based on ground-truth simulation of lagged effect curves representing the delayed relation with excessive zero malaria cases as seen in our study population. The spatial unit used in the simulation was 60 sub-districts in Tak province (*I* = 60). The risk factor *x*_*it*_ was set to represent the weekly series of climatic covariate as *x*_*i*1_~*Normal*(1, 0.01), *t* = 1 and *x*_*it*_ = *ρx*_*it* − 1_ + *ε*_*t*_, *ε*_*t*_~*Normal*(0, 0.01), *t* > 1 with *ρ* = 0.8 and 0.3 to represent two different situations of high and low levels of collinearity, correlation between lagged variables. Collinearity is expected to produce unstable computation and increase uncertainty in model estimates depending on the strength of correlation. Results from a simulation study suggested that even low correlation might decrease accuracies in model parameter estimation [58]. Therefore, we also investigated the model behaviors at different levels of collinearity based on previous research and simulations [58–60]. Then we generated the coefficients, *β*_*l*_, to represent two situations of lagged effects in the simulation study as depicted in Fig. 3. The first curve (Fig. 3 left) was the situation in which the effect increased with a peak in lag at 3 weeks and died out subsequently whereas the effect of the second assumed situation (Fig. 3 right) was an exponential decay over lag periods.

To assess different levels of zero-case percentages, since the overall mean of weekly zero-case percentages during the study period was 76.28% (~ 80%, please see Fig. 1), we set *π*_*i*_ = 0.8 ∀*i* in one simulation scenario to represent the excessive zeros in our data set which was relatively high. However, we also would like to consider another situation with a lower degree of zero inflation in our simulation study and therefore *π*_*i*_ = 0.4 was set in the other simulation scenario. One hundred data sets were then generated and for each simulation replicate we created an outcome series *y*_*it*_ of weekly *P. falciparum* malaria incidence, with time period, *t* = 1*, …,*20 weeks, from a Poisson distribution coupled with relative risk μ_it_. Hence the simulated data were created as *y*_*it*_~ZI − *Poisson*(*μ*_*it*_) which follows

$${\displaystyle \begin{array}{l}{y}_{it}\sim \left\{\begin{array}{c}{\pi}_i\times {1}_{\left\{{\mathrm{y}}_{it}=0\right\}}\\ {}\left(1-{\pi}_i\right)\times Poisson\left({\mu}_{it}\right);{y}_{it}\ge 0\end{array}\right.\\ {}i=1,\dots, 60;t=1,\dots, 20\\ {}\log \left({\mu}_{it}\right)=\log \left(\mathrm{po}{\mathrm{p}}_i\right)+{\delta}_{it}+{\sum}_{l=1}^{10}{\beta}_l{x}_{it-l}\\ {}{\delta}_{it}={\beta}_0+{\upsilon}_i+{\lambda}_t\\ {}{x}_{i1}\sim Normal\left(1,0.01\right)\\ {}{x}_{it}=\rho {x}_{it-1}+{\varepsilon}_t,{\varepsilon}_t\sim Normal\left(0,0.01\right),t>1\end{array}}$$

where the priors were modeled as

$${\displaystyle \begin{array}{l}{\beta}_0\sim Normal\left(0,{\tau}_{\beta_0}^{-1}\right),{u}_i\sim ICAR\left({\tau}_v^{-1}\right),\\ {}{\lambda}_t\sim Normal\left({\lambda}_{t-1},{\tau}_{\lambda}^{-1}\right),t>0;{\lambda}_t\sim Normal\left(0,{\tau}_{\lambda}^{-1}\right),t=0\\ {}{\tau}_{\beta_0}^{-1},{\tau}_v^{-1},{\tau}_{\lambda}^{-1}\sim Unif\left(0,100\right)\end{array}}$$

To compare the performance of different model assumptions, we denoted the unconstrained model without the zero-inflation structure (one-part model) as GLM, regarded as regular generalized linear modeling with Poisson likelihood; the one-part distributed lag model was named as DLM; and zero-inflation unconstrained and zero-inflation distributed lag models were denoted ZIPGLM and ZIPDLM respectively. To fit the models, the coefficients of the unconstrained models, GLM and ZIPGLM, were fitted as *β*_*l*_~*Normal*(0, 100) for all *l* (*L* = 10), while DLM and ZIPDLM were fitted with three sets of degree of freedom for the basis natural spline as ${\beta}_l={\sum}_j^J{B}_{lj}{\alpha}_j$ where *B*_*lj*_ is the element(*l,j*) of a natural spline matrix (degrees of freedom = 3,4,5). The intercept was assumed to have a zero-mean Gaussian distribution whereas the prior of the probability of being at the point- mass zero was assumed to follow. The prior distribution of all precision parameter was defined as ${\tau}_{\beta_0}^{-1},{\tau}_v^{-1},{\tau}_{\lambda}^{-1}\sim Unif\left(0,100\right)$. This allowed the maximum value of variance to be 100 which was reasonably high for random effect terms on the log scale [55–57, 61]. The results were obtained using the WinBUGS software with two chains of MCMC chains and 100,000 posterior samplers were collected after the burn-in part of 100,000.

### Evaluation metrics

To assess the model assumptions under different simulation conditions, five metrics were used to evaluate the performance of models: bias, root mean squared error (RMSE), credible interval (CrI) coverage probability, mean squared predictive error (MSPE) and model information criteria. The first measure was bias computed as the average difference between the simulated (true) mean and its estimate across the simulated datasets in each scenario. This measure is preferred to be near zero. To investigate the estimation uncertainty, we then also calculate the RMSE, summation of the variance of estimation with the bias squared, which was calculated as the squared root of the average squared deviation between the true and estimated means across the simulation replicates*.* The next metric was the coverage probability calculated as the percentage of the credible interval containing the generated value and preferred to have coverage similar to the pre-specified level. In this simulation study we pre-determined the credibility level at 95%. Then the mean squared predictive error was calculated as the squared root of the average squared difference between the simulated data and the predicted incidence from the posterior predictive distribution across the simulation replications*.* The posterior predictive distribution of *y*_*it*_ is in the form of *p*(*y*_*it*_| ***y***) = ∫_Θ_*p*(*y*_*it*_| ***θ***, ***y***)*p*(***θ***| ***y***)*d****θ*** where ***y*** and ***θ*** denote all data and parameters used to fit the model. Finally, the deviance information criterion (DIC) was also used as the global metric for goodness of fit of models. For any sample primary parameter value *θ*^*g*^ for the conditional likelihood, the deviance is $D\left({\theta}^{\mathbbm{g}}\right)=-2\log {f}_{\theta \mid y}\left(\mathrm{y}|{\theta}^{\mathbbm{g}}\right)$ and $\overline{D}$ is the average deviance over the *g* posterior samplers. The effective number of parameters (*pD*) is estimated as $pD=\overline{D}-D\left(\overline{\theta}\right)$, and finally, $DIC=\overline{D}+ pD$. The DIC was calculated under the assumption of single point estimate of the primary parameter of interest which may not be appropriate for mixture modeling as we assumed for zero inflation [29, 62]. Then an alternative is to compute *pDr* as the variance of deviance and define another measure variant as $DICr=\overline{D}+ pDr$.

### Simulation results

**Fig. 4:**
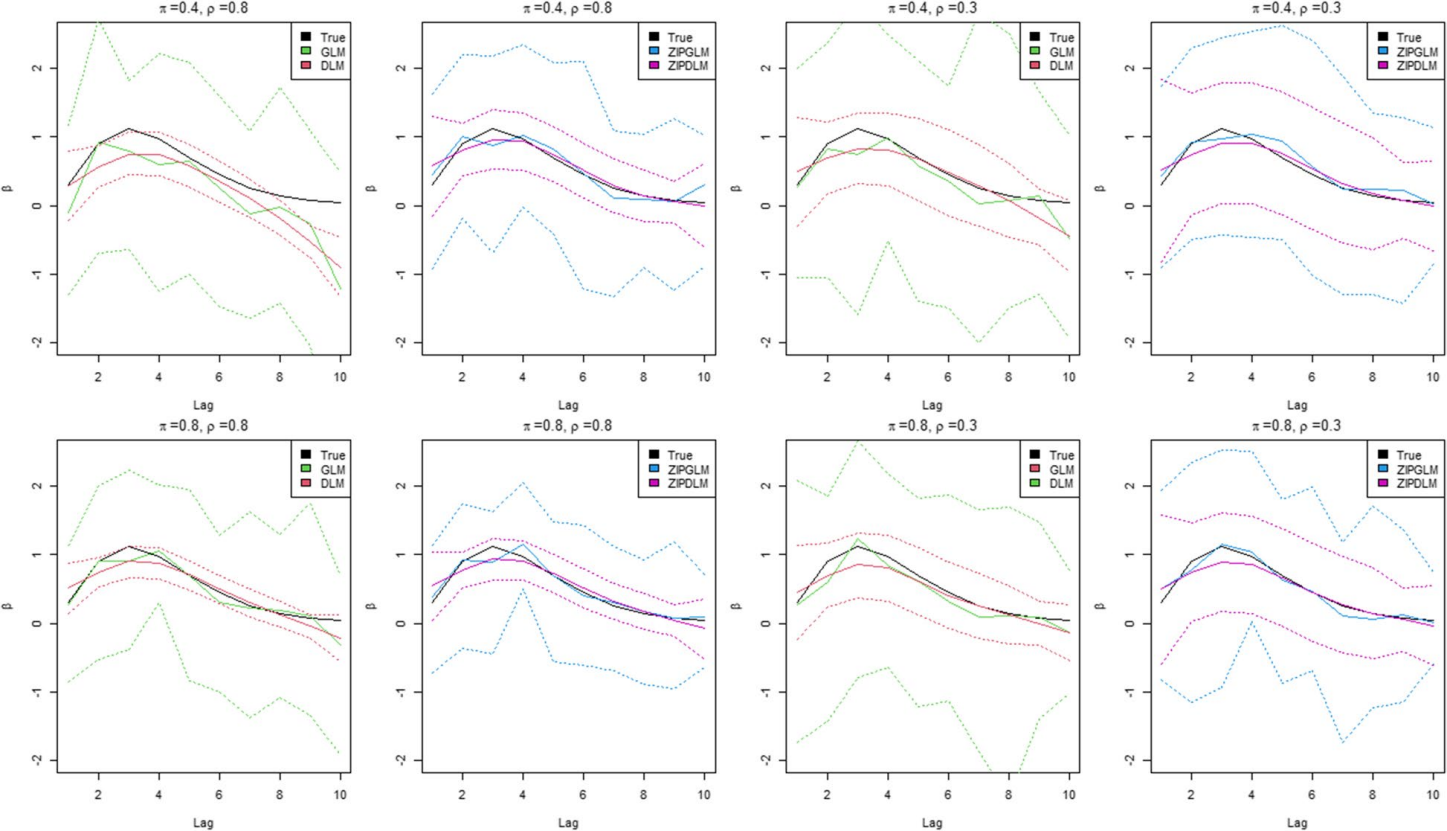
Plots of True and estimated lagged effects, *β*, from the proposed models at different lags (in weeks) under the first simulation scenario in which the effect increased with a peak and died out subsequently. The solid lines are posterior estimates while the dash lines represent the 95% credible band

**Fig. 5:**
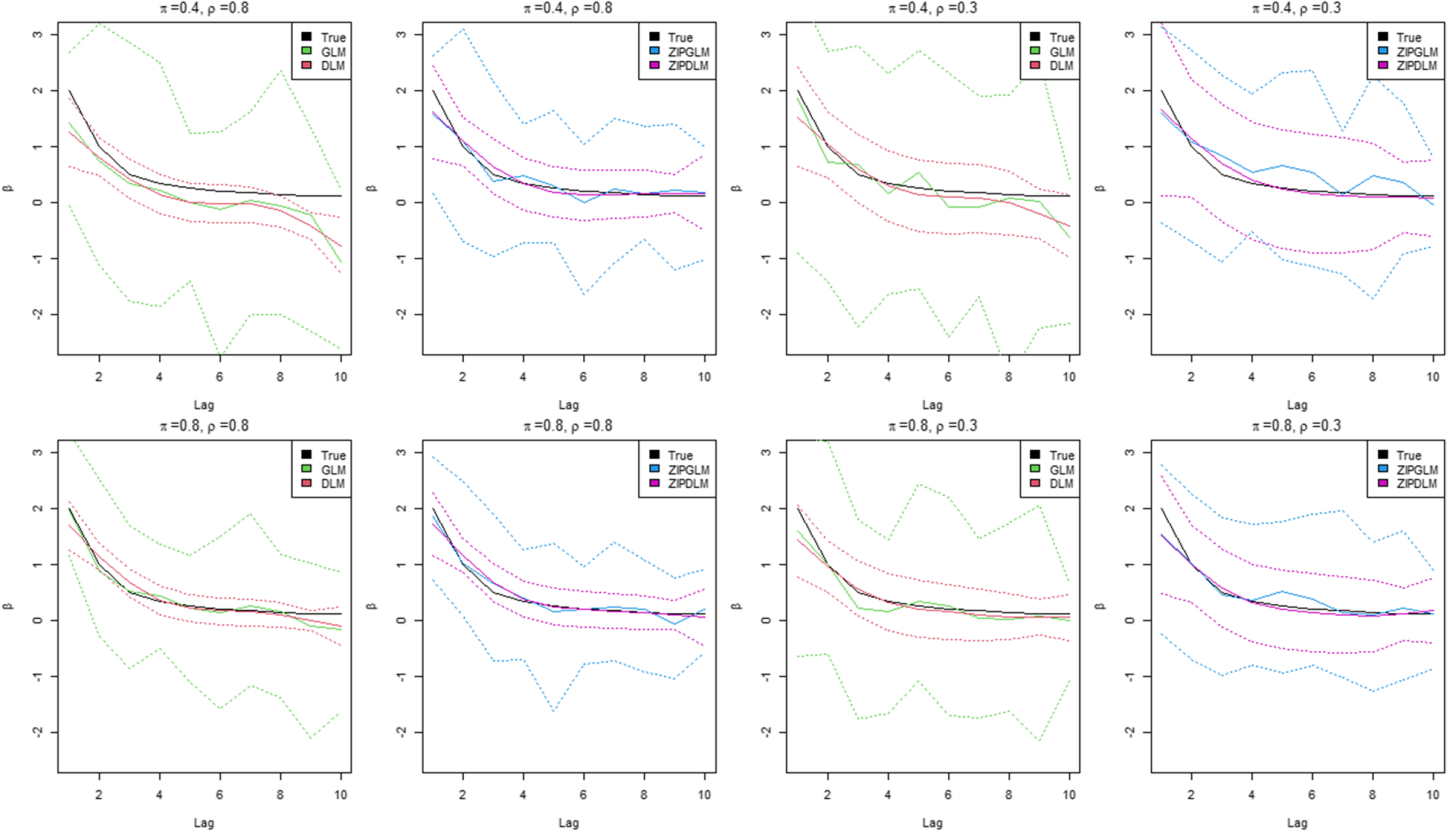
Plots of True and estimated lagged effects, *β*, from the proposed models at different lags (in weeks) under the first simulation scenario in which the effect was an exponential decay over lag periods. The solid lines are posterior estimates while the dash lines represent the 95% credible band

Results of evaluation metrics are shown in Tables 1 and 2. For DLM and ZIPDLM, results were averaged over the spline basis orders. Figures 4-5 represent the estimated lagged effect curves of the true (simulated) coefficients with various simulation scenarios and parameter values. Overall, the distributed lag modeling appeared superior to the unconstrained counterpart, and zero-inflation models outperformed the models which did not account for excessive zeros. All zero-inflation models had similar estimations of zero proportions, *π*. For bias comparison, zero-inflation models had smaller bias while one-part models, GLM and DLM, had negative bias. This suggested that without properly accounting for excessive zeros the estimates can be negatively biased due to the large number of zeros pulling the estimates towards zero. To investigate the variation in estimation, RMSEs were calculated under model assumptions. In general, the ZIP models showed smaller RMSEs than one-part models. Interestingly in situations with high collinearity, e.g. *ρ* = 0.8, the credible bands of DLM appeared to be slightly narrower than ZIPDLM. However, the RMSE does not include only the variance information but is summation of the variance of an estimate plus the square of its bias. Since DLM yielded bigger bias due to excessive zeros, RMSEs of DLM were larger than ZIPDLM.

**Table 1:** Simulation result evaluation metrics from different models in simulation scenario 1

Measure	Model	π = 0.4		π = 0.8	
		***ρ*** = 0.3	***ρ*** = 0.8	***ρ*** = 0.3	***ρ*** = 0.8
**Bias**	ZIPGLM	0.037	0.055	−0.008	0.012
	ZIPDLM	−0.003	0.008	−0.004	−0.006
	GLM	−0.178	−0.438	− 0.093	−0.067
	DLM	−0.091	−0.217	− 0.071	−0.051
**RMSE**	ZIPGLM	0.791	0.881	0.508	0.574
	ZIPDLM	0.487	0.505	0.288	0.314
	GLM	0.942	0.959	0.833	0.875
	DLM	0.552	0.568	0.471	0.485
**CrI Coverage**	ZIPGLM	0.773	0.710	0.764	0.700
	ZIPDLM	0.933	0.799	0.927	0.740
	GLM	0.665	0.522	0.726	0.682
	DLM	0.607	0.484	0.710	0.599
**DIC (pD)**	ZIPGLM	1839.276 (36.599)	1994.511 (37.958)	3577.608 (61.961)	4056.092 (63.363)
	ZIPDLM	1838.029 (36.446)	1991.848 (35.182)	3576.794 (62.771)	4053.807 (61.442)
	GLM	1871.325 (58.264)	2025.734 (48.241)	3610.005 (69.376)	4078.526 (69.081)
	DLM	1868.994 (45.269)	2030.592 (45.277)	3629.755 (68.752)	4076.888 (65.247)
**DICr (pDr)**	ZIPGLM	1839.296 (36.579)	1998.464 (39.911)	3577.627 (61.978)	4059.542 (68.232)
	ZIPDLM	1838.321 (36.223)	1992.372 (35.182)	3576.157 (65.141)	4055.133 (62.772)
	GLM	1881.538 (58.264)	2035.515 (49.288)	3642.345 (81.376)	4085.638 (74.148)
	DLM	1874.314 (50.584)	2040.936 (54.709)	3637.313 (74.097)	4082.538 (72.898)
**MSPE**	ZIPGLM	81.268	160.155	143.496	332.312
	ZIPDLM	81.108	159.774	143.082	315.261
	GLM	82.093	164.001	144.612	315.224
	DLM	81.609	165.531	144.096	313.981
**π**	ZIPGLM	0.399	0.402	0.802	0.797
	ZIPDLM	0.401	0.401	0.802	0.797
	GLM	–	–	–	–
	DLM	–	–	–	–

**Table 2:** Simulation result evaluation metrics from different models in simulation scenario 2

Measure	Model	π = 0.4		π = 0.8	
		***ρ*** = 0.3	***ρ*** = 0.8	***ρ*** = 0.3	***ρ*** = 0.8
**Bias**	ZIPGLM	0.131	−0.052	0.082	0.008
	ZIPDLM	−0.039	−0.021	−0.014	−0.002
	GLM	−0.157	−0.408	−0.119	−0.099
	DLM	−0.175	−0.418	−0.091	−0.081
**RMSE**	ZIPGLM	0.874	0.932	0.727	0.559
	ZIPDLM	0.522	0.608	0.388	0.291
	GLM	1.186	1.124	0.828	0.669
	DLM	0.619	0.702	0.434	0.328
**CrI Coverage**	ZIPGLM	0.712	0.700	0.750	0.740
	ZIPDLM	0.933	0.817	0.923	0.803
	GLM	0.632	0.580	0.755	0.695
	DLM	0.619	0.468	0.756	0.630
**DIC (pD)**	ZIPGLM	1720.445 (35.864)	2012.758 (38.732)	3635.735 (62.453)	3983.145 (69.391)
	ZIPDLM	1718.355 (34.256)	2011.769 (37.748)	3633.122 (60.932)	3969.928 (61.206)
	GLM	1762.11 (48.589)	2058.123 (50.145)	3660.459 (68.444)	4002.552 (74.235)
	DLM	1754.994 (44.321)	2053.159 (46.978)	3658.244 (64.442)	4000.879 (72.926)
**DICr (pDr)**	ZIPGLM	1723.112 (39.782)	2016.447 (42.552)	3638.445 (65.534)	3986.157 (70.763)
	ZIPDLM	1719.562 (35.834)	2012.224 (38.026)	3634.559 (61.952)	3970.251 (62.529)
	GLM	1764.965 (55.123)	2059.624 (53.998)	3672.428 (79.952)	4009.482 (75.992)
	DLM	1762.889 (52.216)	2051.223 (52.217)	3667.393 (73.591)	4005.938 (73.576)
**MSPE**	ZIPGLM	98.299	140.603	196.451	289.122
	ZIPDLM	97.002	140.102	196.112	288.112
	GLM	99.506	140.989	197.125	289.756
	DLM	98.954	140.559	196.478	288.422
**π**	ZIPGLM	0.3976	0.425	0.789	0.789
	ZIPDLM	0.3976	0.425	0.791	0.791
	GLM	–	–	–	–
	DLM	–	–	–	–

From this evidence we should not only evaluate on point estimates but also should compare interval estimation. To assess the interval estimation, we compared models using the coverage probability. ZIPDLM has the nearest coverage probability to the pre-specified level of 0.95 whereas other models yielded relatively lower coverage probabilities. According to the simulation results in Tables 1 and 2, DLM particularly had the least coverage especially under high collinearity and excessive zeros. This might be resulting from the interval estimation of narrow credible intervals combined bias pulling towards zero which made the intervals miss the true values. To evaluate the goodness of fit, we applied the MSPE and information criteria to compare overall model fitting. Although all models had similar MSPEs, ZIPDLM had slightly smaller (better) MSPE. In addition, ZIPDLM also had the best (smallest) DIC and DICr. In general, the ZIPDLM model shows an improved the best result across assessment measures in the simulation study.

## Results

### Case study of lagged effects on sparse malaria incidence in an elimination setting along the Thailand-Myanmar border

In this section, weekly malaria incidence data of Takin 2016 were used to demonstrate the application of the proposed models in the previous section. As a hot spot of malaria incidence sub-districts in Tak along the Thai-Myanmar border were chosen to be a case study to show the performance of the models developed in the previous section. In addition, fine scale subnational data, in this case at sub-district level, have rarely been investigated in previous studies of climate and incidence. All cases had infection confirmed by microscopy (thick and thin films) and rapid diagnostic tests done at hospitals (mostly government hospitals) and/or malaria posts in the study area. Records were put into the national surveillance format and joint into the database of the Bureau of Vector Borne Disease at the Ministry of Public Health by the data management unit of each health facility. Clinical and demographic including gender, occupation, age, residential location, disease onset and treatment date were collated from the surveillance system. Individual data were anonymized for their privacy protection.

The sub-district level population data were extracted from the online public database of the Bureau of Registration Administration. Geographical and administrative unit information were collated from the Ministry of Interior. The updated official geographical information system files at sub-district level were supplied by the Department of Local Administration. Data on average daily temperature, average daily relative humidity and total daily rainfall were considered in this study and collected from the Meteorological Department from five weather stations across the region during the study period. The weather variables were estimated using inverse distance weighted interpolation with tension spline in ArcGIS software version 10.3.1 and aggregated to weekly intervals at sub-district level as total weekly rainfall and average weekly temperature and relative humidity.

**Fig. 6:**
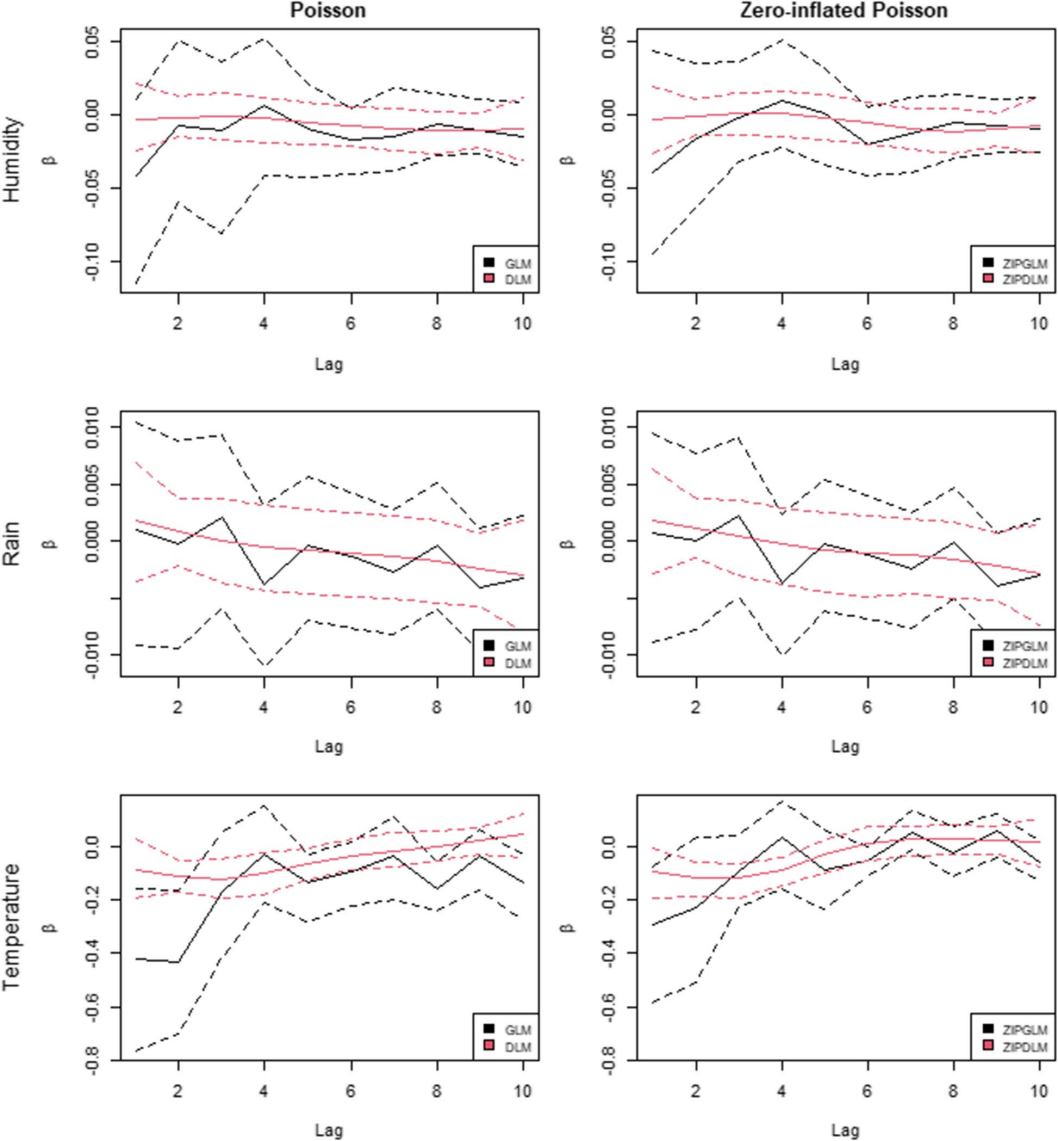
Plots of posterior mean (solid line) and 95% CrI (dash line) of lagged coefficients averaged over degrees of freedom of *P. falciparum* (*Pf*) incidence with climatic factors under different model assumptions

Fig. 6 shows the lag-coefficients between sub-district malaria incidence and climatic factors. The four models in the previous section were used to investigate the shapes of their relationships with the three environmental variables. The distributed lag models, both DLM and ZIPDLM, yielded smaller credible intervals and smoother mean lagged estimates of the coefficients for all weather factors. For humidity, the association started with a slightly negative effect for early lags and had a peak at around weeks 3–4 followed by a slowly declining trend. The relationship with rainfall began with a positive effect and then decreased over time. The association dropped to zero around lag 3 and was negative thereafter. The negative bias in one-part modeling similar to the simulation also occurred in the association with temperature. It could be seen that lagged estimates in GLM had a relatively large negative coefficient which might have resulted from the excessive zeros in our data. So, we focused on the ZIPDLM model for the temperature effect. The trend started negatively and then increased until around week 8 in which the curve remained afterwards. It seemed the proposed models, particularly ZIPDLM producing the best performance across evaluation measures, could be used to analyze the shape of association with malaria incidence useful for future disease control planning. However, additional issues were further discussed in the following section.

## Discussion

This study presents a new method to better quantify the association between climate and malaria over space and time which is suitable for near elimination settings. This can help better understand the impact of different factors on past spatiotemporal trends in incidence and to better predict future trends. It does so by simultaneously addresses the analytical challenges of accounting for the lag between changes in climate and changes in malaria transmission due to mosquito and parasite life cycles and an excess of zero cases in the data encountered in low transmission settings. While count distributions, such as the negative binomial, can add flexibility by incorporating a separate heterogeneity parameter, these distributions do not always ensure adequate fit [43], especially in the case of excessive zeros. Without proper modeling, the high number of zeros tends to lower the mean estimates and neglect of excess zeroes can result in the biased estimation of parameters [44].

To address the analytical challenges, we developed a methodology which accommodates the zero inflation and delayed effects present in our data. In our simulation study, results showed that it was crucial to address both excessive zero cases and lagged nature of disease transmission. The models which did not accommodate for excessive zeros, i.e. GLM and DLM, yielded negatively biased estimates. This is important when planning for disease elimination because the bias can consequently lead to false recommendations and therefore potentially ineffective control activities. On the other hand, the unconstraint model produced high uncertain estimation due to collinearity between lags periods which could mask the significant association. In contrast, addressing only the lag effect could yield too narrow interval quantification. The model which accommodated both issues, ZIPDLM, appeared to have the overall best performance across evaluation metrics and simulation scenarios.

To demonstrate this with real data, we applied the developed framework to a case study of malaria incidence in Tak in which there were malaria hot spots. As suggested by previous studies there may be a temperature range critical for mosquito survivability [63, 64]. In addition, higher temperatures also accelerate multiplication of the *Plasmodium* parasites inside the vectors [14]. Since the peak period of malaria transmission in the GMS is usually in the rainy season which usually has a lower temperature, this could prolong the vector’s longevity in the region and hence the transmission could also occur at longer lags in the GMS. It has also been suggested that the optimal rainfall increases the adult mosquito’s size at early lags of 1 to 2 weeks [65] which was also similar to our finding. In our analysis, it was also found that the strongest associations of malaria with humidity occurred at lags 3–4 and declined thereafter. Increasing relative humidity in an optimal lag period and range has a positive relationship with malaria incidence [66]. On the other hand, rainfall had a shorter lagged effect in our results with negative effect after a few weeks’ lag. Though increasing rainfall can make *Anopheles* larval habitats more receptive [67], flooding due simply to redundantly heavy rainfall, which often occurs in Thailand during the rainy season, may affect the vector’s life cycle. It has been shown that extreme weather can also impact the survival of vectors [68, 69] while light rainfall with suitable amounts of rain may be a more suitable condition for mosquito development.

There are several limitations in our study that should be acknowledged. We only considered three climatic factors (average humidity, total rainfall and average temperature) in association with malaria incidence due to data availability, although a wide range of other climate variables exist [70]. Other environmental variables than climate such as from remote sensing e.g. normalized difference vegetation index (NDVI) and surface water could also be incorporated [71]. Nonetheless, as the malaria parasite is transmitted from human to human via the bite of infected female mosquitoes of the genus Anopheles and the malaria case distribution and dynamics have been found to be closely related to environmental factors, it is sensible to include this nature of the transmission process when available. Epidemiologically there is a lag between changes in environmental and entomological factors and malaria transmission due to both the life cycle of mosquitoes. Hence, we have developed the methodology to deal with the lagged time-varying distribution of the association which can partly account for the fluctuation of the environmental and entomological variates.

Malaria modeling is not an easy task due to the complex nature and difficulties in data collection. Various factors including entomological and population movement factors are important to investigate malaria transmission especially in the GMS region. In addition, the asymptomatic malaria may play important role in particular when malaria transmission comes to a very low level or very close to elimination in which the imported malaria cases may also contribute more than indigenous cases to malaria incidence. However, due to limited data and complex nature of malaria transmission, we could only apply secondary data from the national passive surveillance system in the study. Nonetheless, there is a need for future development to include those factors and the developed platform can also be utilized and extended to include those epidemiological and entomological variables as available to form a more complex relationship with malaria incidence. Therefore, it is important to further consider confounding factors both at individual and population levels in future studies.

Even though there has been extensive research on factors that influence malaria transmission, the estimations that have been made in relation to climate in malaria endemic areas have been inconsistent across different regions [33]. Due to the complex relationship between malaria incidence, gaps in understanding still exist in the underlying processes of the linkage. Despite the limitations due to data availability the models developed here, particularly those accommodating excessive zero cases and lag effects, should provide a practical and useful step forwards in developing methods to examine the influence of different factors on malaria transmission as new data become available.

## Conclusions

In this study, we have developed a methodology which accommodates zero inflation and lagged complexities of the relationship between malaria transmission and weather dynamics. The modeling assumptions were compared and discussed to identify the most appropriate approaches. We also demonstrated proposed models with real data using weekly malaria incidence data at a fine spatial scale. Because of the complexity of malaria transmission, gaps in knowledge still exist in the underlying mechanisms. The developed methodology is promising and may have the potential to help better understand and improve estimates of past and predict future trends in malaria incidence. This could help policymakers to more effectively distribute resources and plan strategies for malaria elimination.

## Data Availability

The data that support the findings of this study were obtained from the Thai Bureau of Epidemiology, Ministry of Public Health, but restrictions apply to the availability of these data, which were used with permission for the current study, and are therefore not publicly available. However, data may be available from the authors upon reasonable request and with permission of the Thai Bureau of Epidemiology.

## Abbreviations

GMS: Great Makong sub-region
DLM: Distributed Lag Model
GLM: Generalized Linear Model
ZIP: Zero-Inflation Poisson
ICAR: Intrinsic Conditional Autoregressive model
MSE: Mean Squared Error
MSPE: Mean Squared Predictive Error
DIC: Deviance Information Criterion
MCMC: *Markov chain Monte Carlo*
RMSE: Root Mean Squared Error
CrI: Credible Interval
NDVI: Normalized Difference Vegetation Index
